# Ceramic Nanofiber Materials for Wound Healing and Bone Regeneration: A Brief Review

**DOI:** 10.3390/ma15113909

**Published:** 2022-05-31

**Authors:** Déborah dos Santos Gomes, Rayssa de Sousa Victor, Bianca Viana de Sousa, Gelmires de Araújo Neves, Lisiane Navarro de Lima Santana, Romualdo Rodrigues Menezes

**Affiliations:** 1 Graduate Program in Materials Science and Engineering, Federal University of Campina Grande, Campina Grande 58429-900, Brazil; gelmires.neves@ufcg.edu.br (G.d.A.N.); lisiane.navarro@ufcg.edu.br (L.N.d.L.S.); 2 Laboratory of Materials Technology, Department of Materials Engineering, Federal University of Campina Grande, Campina Grande 58429-900, Brazil; 3 Department of Chemical Engineering, Federal University of Campina Grande, Campina Grande 58429-900, Brazil; bianca.viana@ufcg.edu.br

**Keywords:** ceramic nanofibers, regenerative medicine, wound healing, bone regeneration

## Abstract

Ceramic nanofibers have been shown to be a new horizon of research in the biomedical area, due to their differentiated morphology, nanoroughness, nanotopography, wettability, bioactivity, and chemical functionalization properties. Therefore, considering the impact caused by the use of these nanofibers, and the fact that there are still limited data available in the literature addressing the ceramic nanofiber application in regenerative medicine, this review article aims to gather the state-of-the-art research concerning these materials, for potential use as a biomaterial for wound healing and bone regeneration, and to analyze their characteristics when considering their application.

## 1. Introduction

The advancement of medicine has led to an increase in the life expectation of the population, bringing with it diseases related to ageing, such as osteoporosis [1], which is the main cause of morbidity, disability, and premature death in the elderly, according to the World Health Organization [2]. Moreover, infections and bone neoplasms have increased significantly in the last decade [3]. These diseases cause severe injuries and bone problems and associated diseases account for half of the chronic diseases of the world’s population over the last 50 years [4]. 

Concurrently, the number of people with diabetes has increased from 108 million in 1980 to 422 million in 2014 [5], with 1.5 million deaths directly attributed to diabetes in 2019 [5]. In the US and Europe, 6.5 and 8 million people, respectively [6,7], are vulnerable to or suffer from chronic wounds, with an estimated treatment cost of over USD 25 billion [7,8,9]. Additionally, the number of people affected by chronic wounds has increased significantly in the last decade [7,9], and it is possible to estimate that, around the world, every 30 s there is a leg amputation, with 85% of them being related to foot ulcers caused by diabetes [10], reflecting the immeasurable social and economic costs that this problem causes to society.

When the subject is about the treatment of bone lesions and wounds, there is a long history about the use of autogenous, allogenic, and xenogenous materials. There is an urgent need to find reliable and more efficient materials for bone repair [4,11] and for the treatment of wounds, especially chronic ones [8,12,13], due to the health, social, and economic problems these diseases cause. These problems have led to a remarkable increase in healthcare system costs and to a reduction in the quality of life of the affected population [14,15].

An excellent alternative to such treatments has emerged through the development of regenerative medicine, which is an multidisciplinary field that aims to restore, treat, and regenerate tissues and, hence, organ functions, by creating a controlled environment that promotes and orients cell proliferation and new tissue growth [16,17]. Although it presents a simple and applicable concept, regenerative medicine presents some challenges in the reconstruction of living tissue from mature cells or stem cells, such as the control of tissue formation in culture media and the search for the development and improvement of materials that are compatible, efficient, and more accessible for tissue repair [18]. Thus, the environment chosen for neotissue growth, differentiation, and cell development is an extremely important element in this area.

In this sense, nanotechnology provides the possibility to produce surfaces, structures, and materials with nanoscale features, which can mimic the natural extracellular matrix (ECM) and favor certain functions, such as cell adhesion, cell mobility, and cell differentiation [16]. Among the possible structures that could replace the natural ECM, the great potential of nanofibers as 3D membranes/scaffolds stands out in regenerative medicine, due to the fact that nanofibers show a high surface area and a highly interconnected porous architecture, which offer a high loading capacity for biological substances and active species, facilitating the colonization of cells in the scaffold and, also, the efficient exchange of nutrients and metabolic waste between the scaffold and its environment [16,19].

In this context, ceramic nanofibers, notably those composed of bioactive glass and glass ceramics, present biological and chemical properties as well as the ability to mimic the hierarchical architecture of the ECM, depicting their great potential applications in the regeneration of bone tissue and wound healing [20,21,22,23]. 

Ceramic nanofibers are biodegradable and present excellent bioactivity characteristics, which favors the formation and deposition of new tissues. These fibers have advantages over several polymeric graft nanofiber materials, such as lower risks of morbidity or infection of the donor site; a high amount of reabsorption capability; ease to sterilize and store; surface nano-topographical features such as nanoroughness and micro- and mesopores that favor cell adhesion and proliferation; surface charge characteristics; and a higher number of surface-active sites. Moreover, 3D sponge/scaffolds or 2D membranes constituted by ceramic nanofibers are easily processed into various shapes and are endowed with suitable properties for the controlled release of therapeutic inorganic ions, in order to promote tissue proliferation and avoid infections; also, they exhibit a highly interconnected porous structure, with a porosity often above 90% and a high surface area, which, according to composition, present the ability to mimic the ECM of the natural bone or the skin. Based on these characteristics, ceramic nanofibers have considerable advantages over polymeric systems for bone regeneration and wound healing [24,25,26].

Moreover, applications of ceramic nanofiber were also observed in other important areas recently, such as drug and gene delivery, stem cell therapy, imaging, and diagnostics [27], which point to the versatility of these materials.

Considerable research has been conducted to explore the properties and applications of ceramic nanofibers, mainly aiming to develop ceramic nanofibrillar scaffolds. In 2006 and 2007 [28,29,30], researchers obtained nanofibrillar scaffolds of bioactive glass (BG) nanofibers and calcium phosphates (CaP), which had chemical–biological characteristics that classified them as a new generation of biomaterials. Recently, Gazquez, et al. [31] produced β-tricalcium phosphate (β-TCP) nanofiber scaffolds with approximately 100 nm in diameter, through electrospinning, offering an excellent platform for bone regeneration studies. Xiao, et al. [32] synthesized hollow mesoporous bioactive glass (MBG) nanofibers via a template-assisted sol–gel method, with an average diameter of around 40 nm. The results indicate that the nanometric diameter and the presence of the mesopores provided the excellent scaffold bioactivity, being considered a promising candidate in the controlled release of drugs and bone tissue engineering.

**Figure 1:**
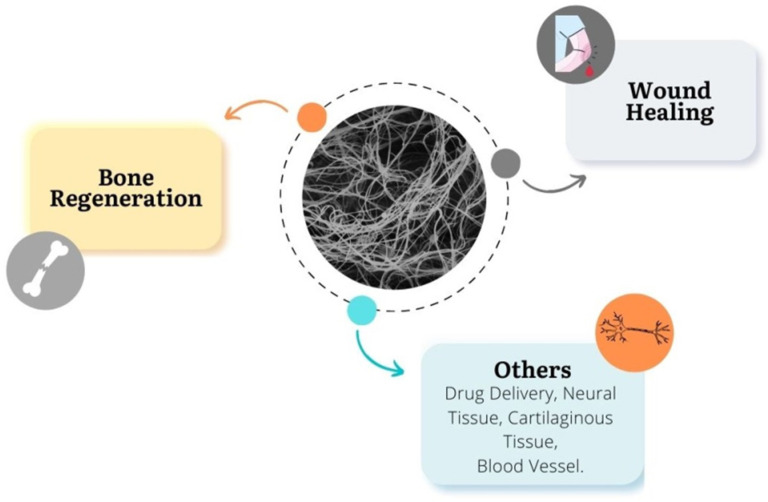
Possible applications of ceramic nanofibers.

In the regeneration of bone tissue and wound healing, the most-used bioceramics are calcium phosphates, silica-based bioactive glasses, and glass-ceramic materials. Figure 1 displays the major biomedical applications of ceramic nanofibers.

Several methods have been developed to produce ceramic nanofibers, such as the template method, in which ceramic fibers are formed using a membrane with numerous tubular pores (5–50 mm thickness) that determines these fibers diameters [33,34]; wet spinning, which consists of pressing a syringe containing the solution through a small orifice (spinneret) into a coagulation bath containing a non-solvent, where the exchange of solvent and non-solvent leads to the thermodynamic instability of the spinning solution, inducing phase separation and precipitation of the solution into a solid fiber [35,36,37,38]; melt blowing, in which molten polymer is extruded through the orifice of a die, the fibers are formed by the elongation of the polymer streams coming out of the orifice by air-drag, and, then, they are collected on the surface of a suitable collector in the form of a web [34,39]; self-assembly, which is a technique where small components are organized in a concentrically manner through non-covalent forces (hydrophobic forces, hydrogen bonding, electrostatic reactions) into ordered and stable nanoscale supramolecular structures or patterns to form nanofibers [19]; phase-separation, in which a gel containing the material precursor solution is cooled to its gelling temperature, and, then, immersed in distilled water for solvent exchange and phase separation due to physical incompatibility. Then, the distilled water is removed and blotted with filter paper, extracting the solvent phase, while the remain phase is transferred to a freeze-drying vessel, creating the nanofiber matrix [33,34]. In the plasma method/technique, normally, a direct current pulse generates a discharge between a pair of metal electrodes in solution giving rise to plasma, which is then expanded and condensed to produce an in situ reaction and the growth of nanofibers [19,40,41,42].

However, the higher yielding methods are centrifugal jet spinning, electrospinning (ES,) and solution blow spinning (SBS). In centrifugal jet spinning, the spinning solution is placed in a rotating spinning head that is continuously fed at a certain flow rate, and, when the rotating speed reaches a critical value, the centrifugal force overcomes the surface tension of the spinning solution, resulting in the ejection of the solution [43,44,45,46,47]. The electrospinning (ES) technique is based on the generation of an electrical field between a solution placed in a capillary tube and a metal collector, and, when the electric field reaches a critical value, electrostatic repulsive forces overcome the surface tension of the polymer solution, producing a charged jet that results in the nanofibers’ formation [48,49]; and solution blow spinning (SBS), in turn, consists of a method in which the spinning solution is pumped through a matrix of concentric nozzles, where the solution passes through the inner nozzle and a pressurized air passes through the external nozzle, simultaneously, then, the high-velocity gas stream overcomes the surface tension of the solution, deforming it, and, during its journey to the collector, the nanofibers are produced by solvent evaporation [20,21,22,23].

These techniques have received great attention, recently, for the production of continuous nanoscale ceramic nanofibers [39,45,50], not only because of their high productivity, compared to the others, but also because of important advantages. ES is a simple, versatile, and efficient technique for obtaining a reproducible ceramic nanometric. SBS provides a higher productivity rate, regardless of the type of solvent, which makes possible the use of green solvents and, also, toxic organic solvents; and centrifugal jet spinning, in turn, has a low cost and high operational safety. Furthermore, nanofibers with different and complex morphology, such as a porous, hollow, or core-shell structure, can be produced with these methods [39,51], expanding the use of these ceramic nanofibers in biomedical applications. Among the materials produced by these techniques, titania, calcium phosphate, alumina, zirconia, calcium silicate, silica, and bioactive glasses can be cited [23,45,48,50,52,53,54,55,56,57].

Nowadays, ES is the most studied, and is used rote, for the successful production of several ceramic materials in 1D morphology. However, ES has some drawbacks, particularly related to low productivity, the use of high electrostatic forces to produce fine fibers, and solvents’ limitations due to the requirement of polar solvents with specific dielectric characteristics. The necessity of high voltages in the process, in the order of several tens of kilovolts, is a very sensitive issue, demanding energy consumption and potential risk.

Considering the outstanding results of studies during the last decade on the use of ceramic nanofibers in bone regeneration and wound healing, as well as the scarcity of systematic reviews on these potential applications, this review article aims to gather information about these materials and analyze their characteristics, when considering their application in regenerative medicine, with an emphasis on wound healing and bone regeneration.

## 2. Wound Healing

The skin is the largest organ in the body, with the functions of protecting muscles, bones, ligaments, and internal organs from external damage, whether biological, chemical, or physical (mechanical). However, its functions are affected by cuts, burns, surgical incisions, and diseases such as diabetes. After its structure is compromised, its function must be quickly restored to ensure the body’s homeostasis [8,58].

Healing usually begins, almost immediately, to avoid the risk of contamination by pathogens. However, in people who have difficulty healing, chronic wounds can be formed. In the initial stages of these wounds formation, gram-positive pathogens, such as Staphylococcus (*S. aureus*) and Streptococcus (*S. pyogenes*), are predominant, being the Gram-negative bacteria, such as Escherichia (*E. coli*) and Pseudomonas (*P. aeruginosa*), observed in the final stages [13,59], in which there tends to be an invasion of the deeper tissues of the skin. Moreover, it should be highlighted that patients with diabetes are more predisposed to infection, notably caused by *S. pyogenes* and *S. aureus* [13,60].

The body responds in different ways to fight the infection, however, when the wound becomes chronic or even acute, the use of wound dressing is necessary, once the antimicrobial action makes it possible to fight infection and stimulate cell growth [12,13]. In this sense, wound treatment dressings must have specific characteristics, such as protecting the wound from the external environment, reducing pain, removing exudate, keeping the moist environment, allowing oxygenation, gas exchange, and fluid passage, and, also, inhibiting the invasion of pathogens. Due to this, they must be preferably porous, contain antimicrobial agents, and have a 3D structure to favor cell growth and adhesion [7,58,61,62].

In this context, nanofibrous scaffolds have been gaining much attention due to outstanding characteristics, such as a high surface area, structure that favors anchoring and cell movement, surface roughness on a nanometric scale that facilitates cell interaction and adhesion, and high porosity, which allows the passage of nutrients and the output of metabolic waste [8,58,63,64]. Therefore, nanofibers are considered ideal dressing materials, as they can mimic the structure of fibrin clots and trap blood platelets in the wound, promoting the deposition, orientation, and maturity of collagen fibers, favoring hemostasis and acting as a physical barrier to avoid the pathogens’ penetration and prevent infection. Additionally, they act as an inductive template to guide skin-cell restructuring and the subsequent infiltration and integration of host tissues [65]. Furthermore, many tissues and organs are similar to highly organized, hierarchical, and nano-sized fibrous structures, which reinforces this trend in scientific research and points out that the development of nanofibrous systems is the new horizon in this technology.

**Figure 2:**
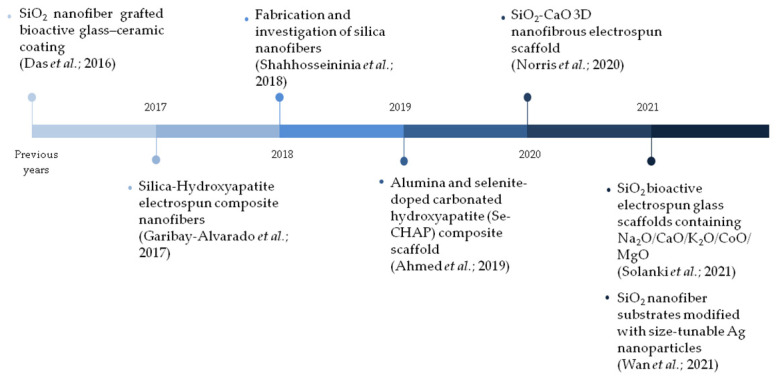
Main studies addressing ceramic nanofibers applied in wound-healing applications over the last six years [20,66,67,68,69,70,71].

Studies have observed [12,13,62] that nanofibrous membranes enable protection against pathogens and control of environmental humidity, favoring cell proliferation and the supply of molecules and bioactive ions and, also, reducing scar formation and healing time. In this context, Figure 2 exhibits, chronologically, the most relevant works that address the use of ceramic-based nanofibrous systems in wound-healing applications from the last six years.

Scaffolds made of ceramic nanofibers exhibit high porosity, high surface area, well-controlled composition, and good wettability and bioactivity [51]. Silica (SiO_2_) is a type of inorganic material widely used for the scaffolds preparation, due to its hydrophilic nature, physical and chemical stability, and good biocompatibility [72]. 

Due to this, SiO_2_ nanofibers has been investigated in biological applications, and relevant results have been reported. Yamaguchi, et al. [73] produced SiO_2_ nanofibers by ES and utilized this material as a substrate for the culture of Chinese hamster ovary cells CHO-K1 (widely used as transgenic cells for the production of substances) and a HepG2 human cell line (normally investigated as in vitro metabolic simulators). The fibers produced showed diameters in the range of 300–500 nm and porosity of 93.2%. The researchers observed a much faster growth and specific functions of hepatocytes per volume of substrates for SiO_2_ nanofibers culture, when compared to the values obtained in HAPS (fiber sheet composed of pulp of hydroxyapatite, a supposedly effective substrate material for CHO-K1 cell culture). 

Based on these results, Das et al. [20] fabricated a bioactive nanofibrous coating of porous SiO_2_ as a structural matrix on an inert glass surface through the ES technique. This system proved to be non-cytotoxic and biocompatible, increasing the surface hydrophilicity and assisted cells proliferation in a short time and with a suitable adhesion for a proper fixation of the implant to the host tissue. In vitro, it acted as a structural scaffold to anchor hydroxyapatite carbonate, supporting and increasing the uniform deposition of apatite and, also, demonstrating its potential to be used as a biological coating on oral implants, when fibroblasts were used in the evaluation. Shahhosseininia, et al. [66] produced bio-inert SiO_2_ nanofibers, via ES followed by calcination. The nanofibers exhibited diameters between 107 to 370 and revealed a desirable growth, the attachment of L929 fibroblast cells, and, also, an adequate flattening with discrete filopodia in the nanofibrous SiO_2_ structure with no evidence of cytotoxicity effect. 

**Figure 3:**
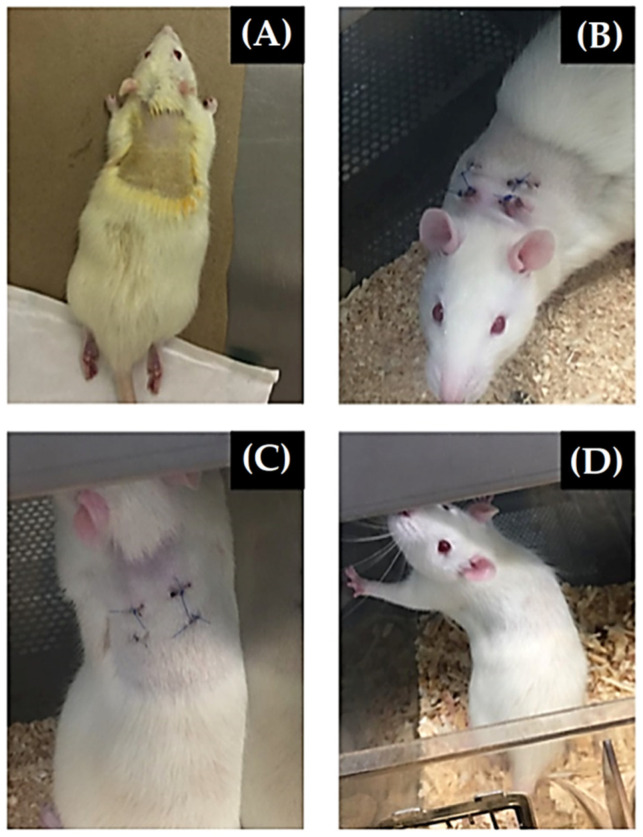
Evolution of the implant area in Wistar rats: (**A**) control rat prior to surgical intervention; (**B**) two weeks after material implantation, being observed inflammation in the incisions on the subcutaneous tissue; (**C**) four weeks of the surgical intervention, with a significant decrease in inflammation; and (**D**) six weeks after the intervention, the rat showed a very noticeable surgical decrease in the incisions’ inflammation, with a considerable growth in the rat’s hair and its scars (reprinted from Garibay-Alvarado et al. [74], copyright (2021), with permission from PloS ONE).

These results corroborate the study by Garibay-Alvarado, et al. [74], who prepared electrospun silica-hydroxyapatite (SiO_2_-HA) ceramic membranes. The fibrillar and porous design had a diameter of 110 nm, a high percentage of viability in a fibroblast lineage, with HA stimulating cell growth and SiO_2_ acting as a support, allowing the cells to anchor. These materials combination improved bioactivity, and no cytotoxicity was observed. In addition, the implant area was monitored in Wistar rats, and a decrease in incision inflammation was observed six weeks after the surgical intervention, as shown in Figure 3. The sutures fell and the rat’s hair grew considerably, covering the scars and indicating the complete healing of the incisions.

**Figure 4:**
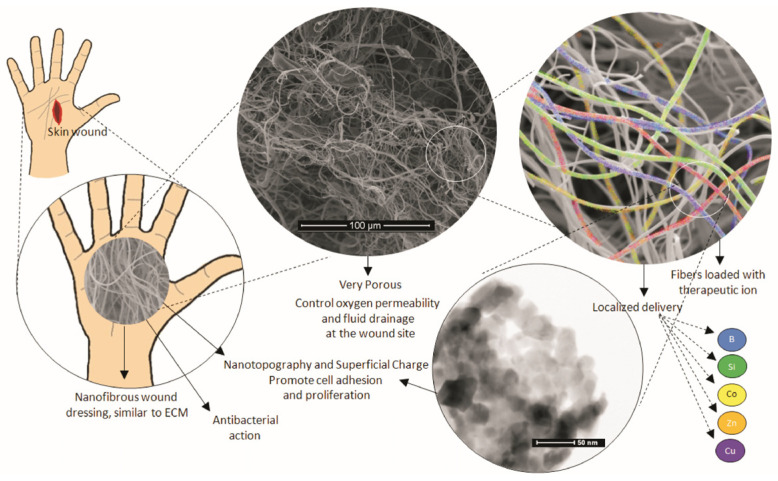
Schematic representation of the influence of ceramic nanofibers on the wound healing process.

Additionally, it is noteworthy that the technology for developing scaffolds/membranes for regenerative medicine has been devoted, in the last decade, to the use of therapeutic inorganic ions (TII), such as zinc (Zn), calcium (Ca), boron (B), strontium (Sr), and magnesium (Mg), which have shown excellent results in terms of anti-inflammatory and antibacterial action as well as cell multiplication stimulation [75,76]. The antimicrobial action caused by inorganic therapeutic ions is becoming more and more necessary in membranes and scaffolds, due to the increase in antibiotic resistance, proving to be a very efficient alternative against Gram negative bacteria, Gram positive bacteria, and fungi [12,77]. Figure 4 presents a schematic demonstration of the influence of the ceramic nanofibers characteristics and the importance of therapeutic ions in the wound healing process.

By loading the engineered scaffold with therapeutic agents, a dual function can be achieved: being a bed for new tissue growth; acting as a carrier for controlled in situ drug delivery; being reported for improved skin penetration, controlled release properties, and protection of drugs against light, temperature, enzymes, or pH degradation,; and stimulating of fibroblast proliferation and reducing inflammation [78,79].

It has been reported that the use of silver (Ag) nanoparticles in biomedical and wound-healing applications, due to non-toxic properties and antibacterial activity, presents the ability to inactivate a variety of Gram positive and negative bacterial strains, without influencing antimicrobial resistance pathways [80]. In this context, Ma et al. [72] prepared SiO_2_ nanofibers through the ES technique and grafted Ag nanoparticles onto the fiber surface through post-treatment, to be used as a reusable wound dressing. The SiO_2_ nanofibers had an average diameter of 260 nm, while 24 nm was the average diameter found for Ag nanoparticles. Their results showed that there was an efficient inhibition of *Escherichia coli* proliferation, with a long-term antibacterial effect, and this inorganic wound covering can be renewed through calcinations without losing its flexibility and antibacterial effect. It has also been shown that nanofibers have no toxicity to human cells and can promote the growth of human cells over a wide concentration range.

Electrospun SiO_2_ substrates modified with size-tunable Ag nanoparticles were also prepared in the work of Wan, et al. [67]. With a diameter ranging from 265–390 nm, these composite nanofibrous substrates have been demonstrated to act as a versatile surface-enhanced Raman scattering (SERS) platform that can perform the label-free detection of bio-macromolecules of bacteria, and, also, possess outstanding antibacterial activities against *S.aureus* and *E. coli*, being possible to be applied as an antibacterial dressing.

Calcium oxide (CaO) nanoparticles can be incorporated into electrospun matrices, in order to achieve improved cell viability and differentiation. Moreover, it has been reported that the possibility to replace antibiotics by the use of alternative antibacterial agents, such as CaO nanoparticles, once this material has demonstrated significant antimicrobial and antifungal activities [81]. Norris, et al. [68] incorporated CaO into a nanofibrous SiO_2_ scaffold produced by ES. The fibers produced with 70% of SiO_2_ and 30% of Ca had an average diameter of 340 nm and a surface area of 43.1 m^2^g^−1^, while the fibers with 80% of SiO_2_ and 20% of Ca had an average diameter of 210 nm and a surface area of 40 m^2^g^−1^. A significant increase was observed in the production of human vascular endothelial growth factor (VEGF), in a human dermal fibroblast cell line (CD-18CO) exposed to the BG samples, and, also, improved wound healing when compared to the control for both compositions.

Although BG are extensively investigated and used for wound-healing applications, the studied glass compositions have already been shown to form a layer of hydroxycarbonate of apatite (HCA) on their surface [69,82]. However, just one layer of HCA can inhibit hemostasis, and Ca deposits can impede the healing of ulcers [69,83,84,85,86]. In this context, Jung and Day [87] produced borate glass fibers scaffolds by the melt blow technique, containing one or more trace elements of Cu, F, Fe, Mn, Mo, Ni, Sr, and Zn, chemically dissolved in the material at a concentration of about 0.05 and 10% by weight. This biocompatible device has shown successful clinical results in healing diabetic foot ulcers that did not heal under conventional treatment conditions.

**Figure 5:**
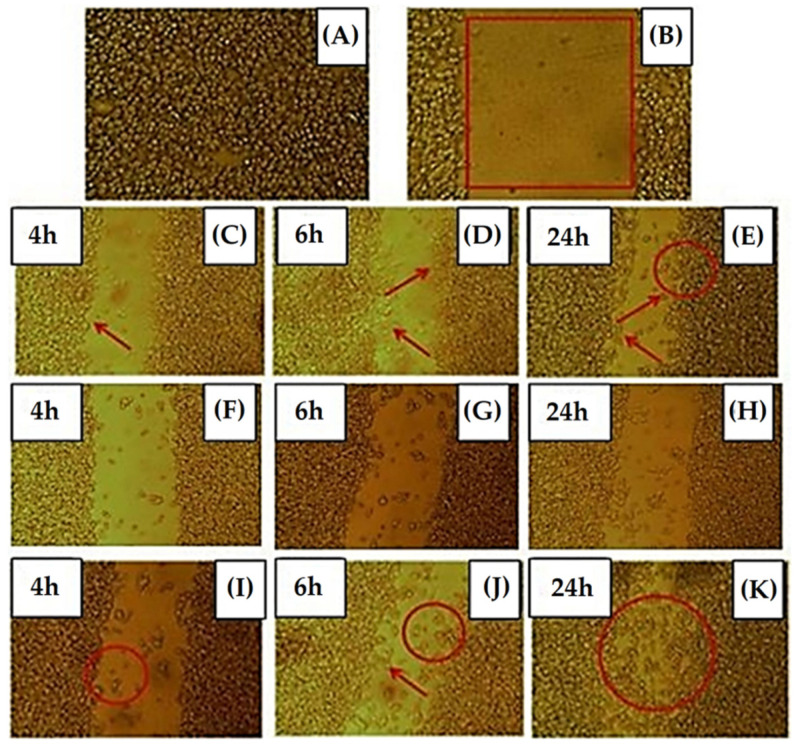
Optical images of an in vitro wound-healing assay undertaken on a human skin fibroblast cell line: (**A**) SV 40-transformed GM 00637; (**B**) scratch created by micropipette on the confluent cell culture plate; (**C**–**E**) control sample at 4, 6, and 24 h, respectively; (**F**–**H**) ABGnf (without boron) at 4, 6, and 24 h; and (**I**–**K**) ABGnf (with boron) at 4, 6, and 24 h, respectively. Reprinted from [88], copyright (2020), with permission from the International Journal of Applied Glass Science.

Also, an in vitro wound-healing assay (Figure 5) Saha, et al. [88] evidenced higher wound-healing rates than the antibacterial bioactive glass nanofibers (ABGnf) of composition 1–2 mol% of B_2_O_3_, 68–69 mol% of SiO_2_, ~1 × 10^−3^ mol% of Ag_2_O, and 29–30 mol% of CaO, after 24 h of testing, with 82% and 65% wound-healing rates for ABGnf, respectively, against a wound-healing rate of 47% measured for the control group. The enhanced cell proliferation observed for ABGnf in the Bo-treated group may be attributed to an increased production of tumor necrosis factor (TNF α) and interleukin-6 (IL-6) response, which, subsequently, rises VEGF production, and the increment of this growth factor—also, bFGF and their receptor proteins—accelerates endothelial cell migration, a major process of angiogenesis. 

The in vitro cytotoxicity assay undertaken on human skin fibroblast cell line (SV 40-transformed GM 00637) in this study [88] evidenced a cell viability of 97% at 24 h and 95% at 72 h of ABGnf, when compared to the control, indicating that the produced ABGnf has no significant cytotoxic effect over this cell line.

A probable mechanism of the wound-healing potential of Bo containing ABGnf includes mimicking the structure of the fibrin clot, which facilitates the entrapment and aggregation of the platelets. In addition, the Ag presence provides an antibacterial potential to the wound bed, while the dissolution of the ionic products of ABGnf enhances wound healing through growth factors and collagen fiber secretion deposition [88].

Solanki et al. [69] developed a SiO_2_ bioactive electrospun glass scaffold, containing Na_2_O/CaO/K_2_O/CoO/MgO, reporting a sustained rate of delivery for pro-angiogenic cobalt ions, which could be mediated by the Mg content of the glass. The dissolution products stabilized HIF-1 α and induced a significantly higher expression of VEGF, suggesting that the composites activated the HIF pathway to stimulate angiogenesis.

Among the bioceramic systems to wound healing, there are, also, calcium phosphate-based materials. Hydroxyapatite (Ca_10_(PO_4_)_6_(OH)_2,_ HA) is a bioceramic that can be obtained through a variety of chemical reactions, such as hydrolysis or the sol–gel method, and has been, recently, used in a variety of biomedical applications, such as drug-delivery devices and tissue-engineering scaffolds [89]. Although brittleness is one of the main problems with the use of HA, the composite fabrication is able to improve the mechanical characteristics of this material, as it is possible to highlight the use of silicate to act as a reinforcement component in flexible membranes [70].

HA has a relatively open and flexible crystal structure that can accommodate different ionic species to achieve the desired properties, a strategy that has been widely used, since this compound itself does not exhibit antimicrobial properties, is fragile, and has limited contact with host tissue [71]. In this sense, the incorporation of therapeutic metal ions to this material can promote antimicrobial activity.

As already reported in the literature, Cu^2+^ ions have demonstrated an interesting role in wound-healing applications, when compared to growth factors, due to its low cost, high stability, and better clinical safety, increasing angiogenic response [90,91,92]. Moreover, it has already been observed [93,94,95] that Cu^2+^ can stimulate the expression of pro-angiogenic factors, such as growth factor-β (TGF-β) and VEGF, in wounds created in diabetic mice.

Zhao, et al. [96] produced electrospun dressings of bioactive borate glass fibers (6Na_2_O, 8K_2_O, 8MgO, 22CaO, 54B_2_O_3_, 2P_2_O_5_; mol%) doped with CuO (0–3%). Fibers exhibited diameters ranging from 0.4–1.2 μm and, after immersion in SBF, induced the HA layer formation in nearly seven days. Cellular tests showed non-toxicity to human umbilical vein endothelial cells (HUVECs) and fibroblasts, promoting HUVEC migration, tubule formation, and vascular endothelial growth factor (VEGF) secretion. Moreover, at 7 and 14 days post-surgery, fibers doped with 3% Cu showed a significantly better ability to stimulate the expression of HUVEC genes related to fibroblast angiogenesis, when compared to undoped fibers and untreated defects (control).

Table 1 summarizes the main characteristics about ceramic nanofibers with promising application for wound healing, showing information about their most relevant biological properties according to the group of ceramic nanofibers mentioned throughout the manuscript. 

## 3. Bone Regeneration

The number of diseases and bone fractures has been growing worldwide, due to ageing and an increase in population weight problems, such as obesity [97]. Due to this, bone injuries, notably “critical defects” (which are large bone defects that are not able to regenerate on their own [4]), whether resulting from trauma, infections, or tumors, have become a complex challenge for current orthopedics [97], bringing great losses to health and life quality.

In this scenario, it is estimated that there are more than 4 million bone tissue transplantations annually, the second-most performed in the world [3,98]. While autogenous bone grafting is still considered the “gold standard” for repairing bone defects [4,98], however, its disadvantages includes secondary damages such as high morbidity at the donor site, infections, pain, shape and size limitations, and insufficiency of autogenous bone, among others [4,98].

This awakens the need of search for alternative materials for bone transplantation and reconstruction purposes, standing out the development of 3D scaffolds systems for bone tissue engineering, which are biocompatible, biodegradable, and favor cell adhesion and proliferation. In this sense, scaffolds made of ceramic nanofibers displays interesting characteristics, such as nanorugosity, nanotopography, wettability, bioactivity, and ECM-like morphology, which favor cell multiplication and have shown promising results for the repair and rebuild of damaged bones, including critical defects [99,100].

Ceramic materials used for bone regeneration purposes are known as bioceramics, which can be classified as bioinert, bioactive, or bioresorbable, and display effective properties for the use in scaffolds. Bioinerts maintain their physical and mechanical properties after implantation, exhibiting minimal interaction with the surrounding tissue, high chemical stability, and mechanical resistance, with alumina and zirconia being the most common materials in this subcategory [101]. Bioactive ceramics have the ability to settle on the surface of the implant, allowing a deep interaction and chemical bond with living bone tissue, without the intervention of the fibrous tissue layer. Bioresorbable ceramics, in turn, are gradually degraded or absorbed in vivo, replacing the damage site with the new tissue formed.

Several ceramic materials have been used in scaffolds for bone regeneration purposes, being able to increase cell proliferation and/or with antibacterial action and/or aiming to increase resistance in hybrid systems, such as: CaP [98,102], MgO [103,104], BG [98,105], calcium silicates (CaSi) [3,106], Mg_2_SiO_4_ (fosterite) [107], TiO_2_ and Na_2_Ti_6_O_13_ [108,109], perovskite ceramics [110], γ-Fe_2_O_3_ and Fe_3_O_4_ [111,112], etc. Among these, CaP, BG, and CaSi stand out as the most commonly bioactive and bioresorbable ceramics for applications in bone regenerations [113].

**Figure 6:**
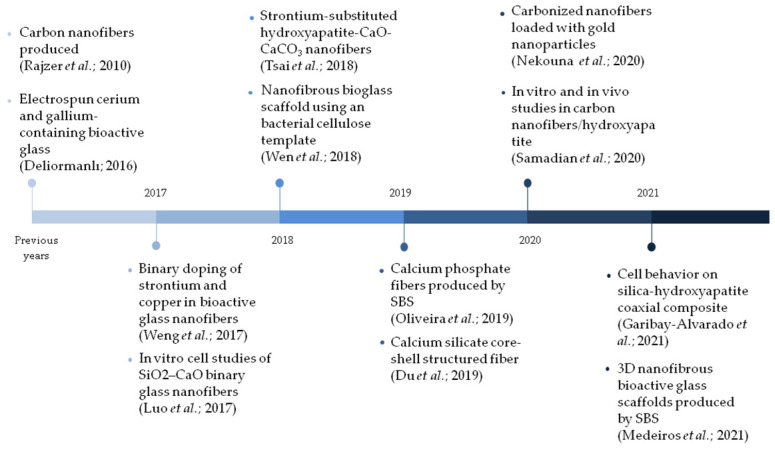
Main studies addressing ceramic nanofibers applied in bone regeneration applications over the last six years [23,55,56,74,114,115,116,117,118,119,120,121].

On the other hand, recent studies have shown that bioceramic nanofibers exhibit an outstanding performance, when compared to powdery or micrometric 1D materials for the use in bone tissue regeneration. In this context, Figure 6 covers, chronologically, the most relevant works that address the use of ceramic-based nanofibrous systems in bone regeneration applications over the last six years.

The literature indicates an association between the inherent characteristics of the nanometric character of the nanofibers bioceramics microstructure (fiber topography, arrangement/disposition of nanofibers, pore sizes and distribution, etc.) and the chemical-biological properties of these materials, generally with a synergism between them, which implies the extremely satisfactory performance in cell adhesion, proliferation, and differentiation in the in vitro and in vivo assays of the nanofibrous scaffolds [28,55,122,123,124,125,126,127,128].

CaP-based bioceramics, such as HA, β-TCP, Ca_3_(PO_4_)_2_, and biphasic calcium phosphate (BCP), a mixture of HA and β -TCP composed of the same ions as bone, are inorganic materials that show excellent biocompatibility and have received great attention for bone repair applications, due to their chemical and structural similarities with the inorganic phase of human bone. They have also been shown to be efficient bone substitutes that respond well to material resorption/bone replacement events, being widely used in the hard tissue replacement area, as well as being used in various biomedical applications aiming at bone regeneration [129]. Among them, β-TCP and HA are the most used materials for bone regeneration, since they have a similar composition to the inorganic constituents of bone, allowing the production of apatite, the main inorganic bone component. When compared to β-TCP, HA is slowly reabsorbed and undergoes a little conversion to a bone-like material after implantation. However, β-TCP scaffolds generally exhibit lower strength than HA scaffolds with the same porosity, which makes their use in bone repair challenging [130,131,132,133].

Holopainen and Ritala [122] produced HA fibers through the ES technique, followed by annealing (electroblowing), and observed that fibrous membranes had fiber mean diameters ranging from 200 ± 70 to 330 ± 140 nm, depending on the solution characteristics and the experimental parameters used. The relative humidity (RH) chosen for spinning was an important factor for the fiber properties, with it being noticed that a RH greater than 25% promotes an increase in the amount of wet droplets that reaches the collector, hampering the collection of continuous fibers, while smaller RH values, generally, induce the formation of smoother and larger diameter fibers. In spinning techniques like this, as well as in ES and SBS, the RH is a parameter that is difficult to control, once it is related to the climatic characteristics of the environment where the spinning takes place. It was also shown that randomly oriented HA fibers induce the fast formation of a homogeneously apatite layer around the fibers after a 6 h immersion in simulated body fluid (SBF), being considered a high bioactivity material. In the same year, Yi, et al. [134] developed porous HA fibers loaded with bovine serum albumin (BSA) that exhibited good drug-controlling release properties, observing that, after three days of immersion in phosphate buffered saline solution (PBS), there was an abundant formation of nano-bone apatite on almost all surfaces of the glass fibers.

The β-TCP has also shown excellent performance as a scaffold for bone regeneration, promoting osteogenic induction and biosorption [135,136,137]. Gazquez et al. [31] presented the first report of fabrication of β-TCP fibrous scaffolding using ES. The fibers showed small diameters, in the range of 100–125 nm, after calcination at 950 °C and unidirectional grain growth during the sintering phase, with the smallest grain size ever produced. They noticed that fast heating/cooling and short sintering times help to keep small grain sizes, producing a three-dimensional material that can provide an excellent platform for bone regeneration studies. Calcination temperatures were also analyzed by Oliveira et al. [56] in the production of biphasic HA and β-TCP submicron fibers, using the SBS spinning technique. Fibers were calcinated at 900 and 1000 °C, proving to be non-cytotoxic, presenting inhibitory concentration (IC) > IC50, and, also, exhibiting a formation of acicular apatite layers after immersion in SBF.

However, CaP nanofibers are brittle and need to be used in combination with some reinforcement component for load-bearing applications. In this sense, Garibay-Alvarado et al. [74] studied the effect of the SiO_2_ and HA combination through the production of coaxial composite nanofibrous membranes of SiO_2_-HA by the ES technique. The obtained blanket showed a nanofibrous characteristic with an average diameter of 110 ± 17 nm after heat treatment at 800 °C, surface area of 6.57 m^2^/g, and a pore size of 15.75 nm. It was possible to observe that the combination of SiO_2_ and HA significantly improves bioactivity, when compared to pure SiO_2_ and HA membranes. In addition, the fibrous and porous design demonstrated a high percentage of viability in a fibroblast cell line, with greater cell viability for the SiO_2_-HA compound, which may be related to the HA ability to stimulate cell growth [138] and the SiO_2_ ability to provide support for cell anchoring [139]. On the other hand, according to the literature, the fibrous architecture helps to maintain a normal phenotype of cells, which plays a fundamental role in the regulation of cell behaviors, such as cell adhesion, cell viability, and proliferation [140].

BG are biocompatible, bioactive, and osteoconductive materials that have been commonly used for bone tissue regeneration [141]. The bioactive capacity of these glasses in bone regeneration applications is directly related to the biological capacity of forming an active layer of HCA, once in contact with biological fluids [142,143,144]. Many studies shows that BG promotes enzymatic activity [145,146] and vascularization [147,148], as well as maintains osteoblastic adhesion, in addition to regulating the growth and differentiation of mensenchymal cells into osteoblasts [149], while, also, exhibiting excellent biocompatibility properties, as observed in in vivo studies [28,150].

**Figure 7:**
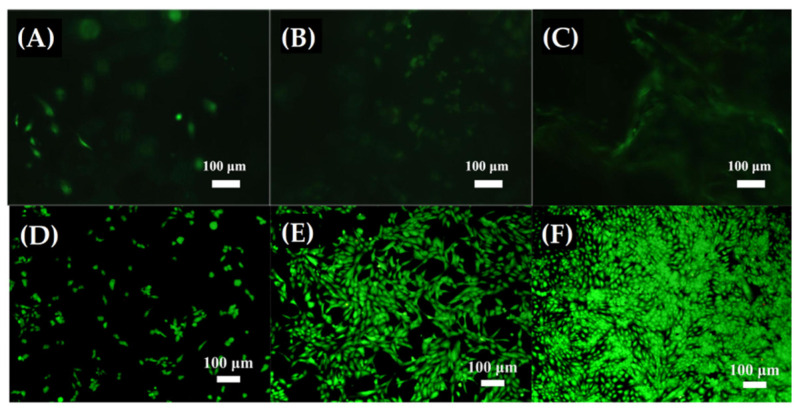
Live/dead immunofluorescence staining results of osteoblast cells cultured for one (**A**,**D**), three (**B**,**E**), and five (**C**,**F**) days on binary glass nanofibrous scaffold (**A**–**C**), as well as a blank control (**D**–**F**). Reprinted from Luo et al. [55], copyright (2017), with permission from Materials Science and Engineering: C.

Luo et al. [55] fabricated nanofibrous 3D binary bioactive glass scaffolds (SiO_2_–CaO) by combining the template-assisted sol–gel technique and using bacterial cellulose as a calcination model. The study confirmed that the Ca/Si molar ratio and the nanofibers diameter can be controlled by the immersion time in the solution of tetraethyl orthosilicate and ethanol. The best results were obtained for the scaffold constituted of 60 a.% of Si and 40 a.% Ca (after 6 h of immersion), which exhibited a nanofiber diameter smaller than 29 nm, with a highly porous structure and a surface area of 240.9 m^2^g^−1^. As shown in Figure 7, cell differentiation was analyzed by an immunofluorescence-staining assay against a blank control, showing that the binary scaffold induces cell differentiation during five days of culture, with no dead cells being observed. Furthermore, Thiazolyl Blue Tetrazolium Blue (MTT) analysis indicated that cells are viable and proliferate well, as well as that cell viability was significantly higher in the BG scaffold when compared to the control. The excellent biocompatibility, better cell proliferation, and high *alkaline phosphatase* (ALP) activity may result from the combination of nanotopological surface characteristics, macro and mesoporous structure, large surface area, and 3D biomimetic architecture, in addition to the chemical structure that promotes better cell adhesion and Si^4+^ and Ca^2+^ release, which can accelerate osteoblast proliferation and differentiation [151,152,153].

**Figure 8:**
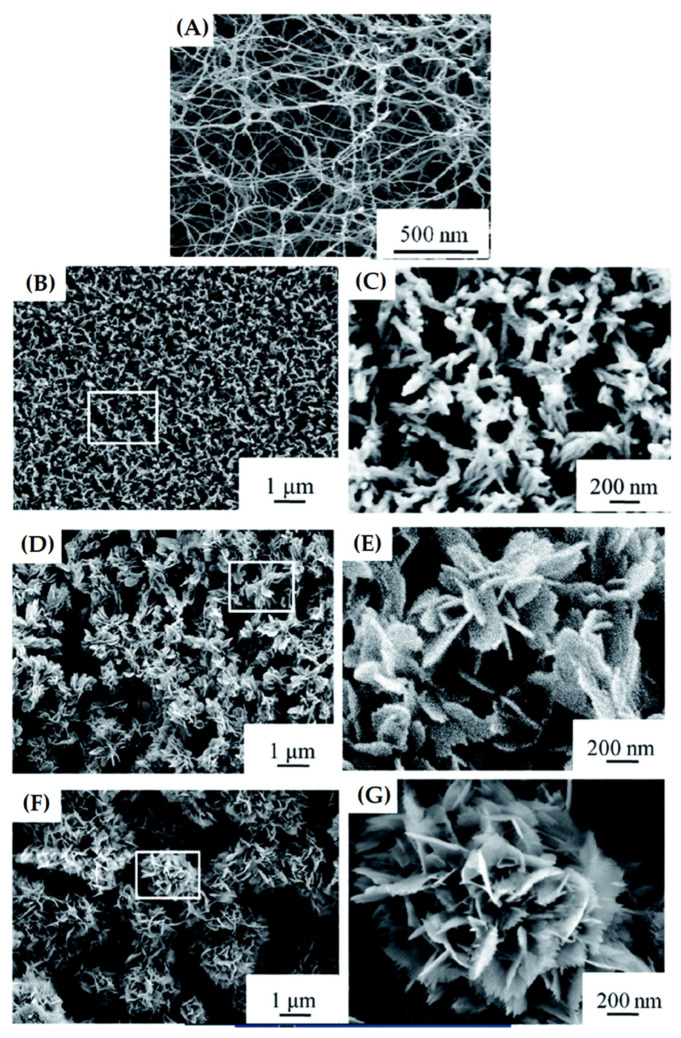
SEM images and the corresponding high magnification images of NBG scaffolds (**A**), after immersion in SBF for (**B**,**C**) one, (**D**,**E**) three, and (**F**,**G**) seven days (insets show local enlarged areas). Reprinted from [156], copyright (2018), with permission from RSC Advances.

Luo, et al. [154] produced a nanofibrous bioactive glass scaffold via a sol–gel route, using a 3D bacterial cellulose aerogel as a template, followed by calcination. Nanofibers exhibited diameters nearly 16 nm, with an interconnected porous structure that proved to be highly bioactive. Moreover, in another study, Luo, et al. [155] found similar results for 58S BG scaffolds produced using bacterial cellulose (BC) as a template, which also exhibited bioactivity and biocompatibility with mouse primary osteoblastic cells, as indicated in in vitro cellular studies. Wen, et al. [156] first reported the use of amino-modified BC as template to prepare a 3D nanofibrous BG scaffold, via a modified sol–gel under ultrasonic treatment. Results indicated that the amino groups in the BC template can effectively promote the absorption of the CaO and SiO_2_ deposited through their precursors, promoting the successful formation of the nanofibrous BG scaffold after calcination at 700 °C. The obtained scaffold exhibited an average nanofiber diameter of 20 nm and an interconnected porous structure (Figure 8A). The SBF immersion test showed a deposition of HA on the scaffold surface with an HA morphology varying from a needle-like structure to a flower-shaped structure after immersion times between one and seven days, as shown in Figure 8. The researchers suggested that the rapid formation of HA may be related to the solubility of the nanofibers in SBF solution, which promotes the release of large amounts of Ca^2+^ ions during the initial immersion stage and increases the relative saturation of HA. Xiao et al. [32] also produced hollow mesoporous bioactive glass (MBG) nanofibers via a template-assisted sol–gel method, which exhibited diameters of around 40 nm, with a large specific surface area of 579.0 m^2^g^−1^ and outstanding bioactivity.

**Figure 9:**
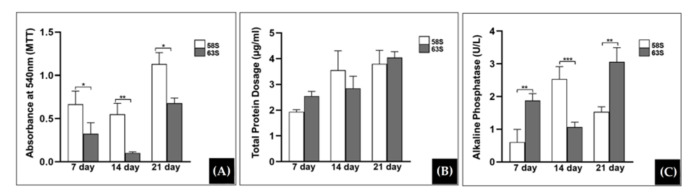
(**A**) MTT activity of MC3T3-E1 cells cultured on studied scaffolds for 7, 14, and 21 days. (**B**) Total protein content (μg/mL), up to 7, 14, and 21 days of cell culture of the osteoblastic lineage MC3T3 cultured on the studied scaffolds. (**C**) Alkaline phosphatase activity (U/L) of MC3T3-E1 cells on the studied scaffolds. (* statistical significant differences with *p*-value < 0.05; ** statistical significant differences with *p*-value < 0.01; *** statistical significant differences with *p*-value < 0.001). Reprinted from [23], copyright (2021), with permission from Ceramics International.

A BG nanofibrous scaffold bioactivity was also studied by Medeiros et al. [23]. In this study, 3D nanofibrous scaffolds of 68S and 63S BG were fabricated by SBS in a one-step process. After calcination at 800 °C, the fibers showed cylindrical morphology with an average diameter in the range of 344 to 358 nm. The high bioactivity in SBF was evidenced with the formation of HA crystals after 12 h of immersion. The MTT assay showed an increase in cell uptake after the culture time, promoting high cell proliferation (Figure 9A). Protein analysis also showed a significant increase in the amount of protein over time (Figure 9B). ALP activity increased after the culture time, exhibiting ALP differentiation levels consistent with cytocompatibility. The scaffolds with the highest presence of Ca showed higher ALP at 14 days, which may indicate that higher Ca dissolution rates induce the proliferation and differentiation of osteoblastic cells (Figure 9C). The smallest amount of Ca ion, in turn, delayed the ALP development, reaching the maximum value after 21 days.

Ceramics based on CaSi, such as wollastonite (CaSiO_3_, CS), larnite (β-Ca_2_SiO_4_), and α-calcium disilicate (Ca_2_SiO_4_), in turn, have shown high biocompatibility and mechanical strength and, also, demonstrate excellent bioactivity and a higher degradation rate than CaP ceramics [3]. The degradation of CaSi releases Si and Ca ions, which promote cell proliferation [114,157]. Furthermore, it is observed that CaSi cements induce in vivo bone formation [158,159], acting as a basis for cell adhesion and promoting cell proliferation and bone tissue growth.

Studies [160,161,162,163] have shown that the chemical components released by CS can stimulate osteogenic proliferation and the differentiation of bone marrow stem cells (BMSCs) and osteoblasts, in addition to exhibiting faster bone regeneration capacity and inducing better angiogenesis when compared to traditional CaP bioceramics.

Lin, et al. [164] produced bioceramics constituted by CS nanofibers, with a hydrothermal synthesis route and calcination step at 800 °C. The nanofibers exhibited a fiber a diameter between 10–30 nm and a high flexural strength by pressureless sintering. The flexural strength test showed that the CS bioceramics reached an upper limit value similar to that of human cortical bone (145.70 ± 2.74 MPa). Bioceramics composed by nanostructured materials can present high densification when compared to micro-sized powders [165], which could explain the high strength obtained by the authors. On the other hand, the bioactivity performed by SBF immersion indicates that this material induces the fast deposition of the apatite layers, which plays an important role in bone bonding between the bioactive material and the host tissue [166,167,168]. These results corroborate with the in vivo experimental results presented in the literature, suggesting the potential application of these CS bioceramics as filler materials for bone implants [160,161,169]. 

**Figure 10:**
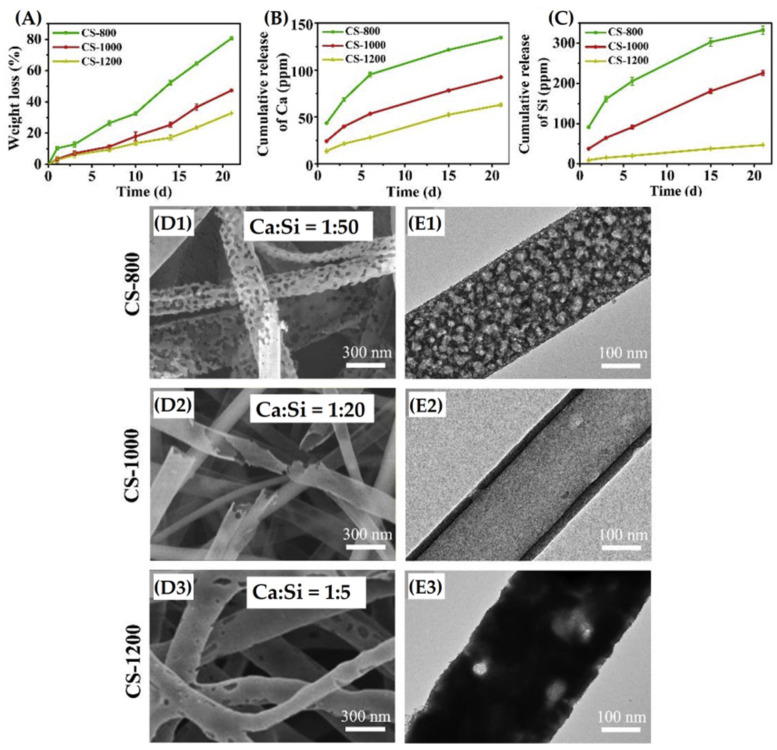
(**A**) Weight loss, cumulative release of (**B**) calcium ions and (**C**) silicate ions from sintered nanofibers of CS-800, CS-1000, and CS-1200; (**D1**–**D3**) shows SEM images and (**E1**,**E3**) TEM images of the corresponding calcium silicate nanofibers, after degradation in deionized water at 37 °C for 21 days; (**D1,E1**) corresponding to CS-800 composition; (**D2,E2**) corresponding to CS-1000 and (**D3**,**E3**) to CS-1200. Reprinted from [170], copyright (2019), with permission from Ceramics International.

More recently, Du, et al. [170] produced CS nanofibers with a core-shell structure, via ES and calcination at 800 °C, 1000 °C, and 1200 °C. Nanofibers calcined at 800 °C presented a higher porous-like structure than fibers fired at 1000 °C and 1200 °C, after 21 days of immersion in deionized water at 37 °C. This may be related to the calcination temperature of the sample, which promotes a faster degradation rate for the sample calcined at 800 °C, as can be seen in Figure 10. The nanofibers showed a faster degradation rate in the core (CaSiO_3_), when compared to the shell (SiO_2_), notably the 1000 °C and 1200 °C fired fibers, which allows, according to the authors, their application as a nanotube drug carrier to provide the controlled release of bioactive ions. In order to investigate the influence of calcination temperature on the microporous characteristics of CS fibers and their ability to induce bone regeneration, Du et al. [128] produced CS nanofibers via ES with sintering at 800 °C, 1000 °C, and 1200 °C. They observed that the rise in temperature promoted greater crystallinity and a lower degradation rate, and that nanofibers calcined at 1000 °C exhibited the better release profile for osteogenic differentiation and the proliferation of mesenchymal bone marrow stromal cells.

Many types of nanofibrous composites have been produced, with the aim of mimicking the natural extracellular bone tissue matrix [115,171,172,173,174]. In recent years, hybridized carbon nanofibers (CNFs) containing inorganic nanoparticles have been reported as materials with great potential for bone tissue repair [54,175]. When compared to organic–inorganic nanofibers, CNF hybrids have distinguished characteristics for bone repair, as they favor the fixation and proliferation of bone cells, such as osteoblasts and bone mesenchymal stromal cells (BMSCs). Moreover, when obtained by heat treatment of the polymer precursor at temperatures below 1000 °C, they integrate into tissues and can undergo a slow oxidation in the biological environment, becoming non-toxic organic forms for the body [176]. In addition, the physical–chemical modification through the incorporation of osteoinductive bioceramic nanoparticles makes the CNF hybrids more flexible and osteocompatible [54].

BG nanoparticles have been used to produce hybridized CNFs, due to their well-known high bioactivity and osteoinductivity. Cheng et al. [54] prepared hybrid CNF/BG nanofibers through ES with different molar ratios between Ca/P, in order to regulate their chemical structures and biological properties. The hybrid nanofibers had an average diameter ranging from 220–320 nm and were capable of inducing the nucleation and growth of apatite with the dissolution of BG nanoparticles. Cheng et al. also observed that scaffolds composed of BG (Ca/P = 1.0) exhibited the fastest proliferation rate and the highest expression of alkaline phosphatase activity. Such results support the theory that cells tend to preferentially adhere to rough surfaces rather than smooth surfaces, promoting better cell proliferation by absorbing bioactive components from the culture medium [116,177,178].

Smolka, et al. [179] noticed that carbon nanofibers containing silicon and Ca compounds exhibited higher HA deposition, after three days of immersion in SBF, when compared to unmodified carbon nanofibers. The nanofibers produced by ES showed a porosity between 0.47 and 0.76 and an average diameter around 190 nm. The carbon nanofibers containing Si and Ca in contact with osteoblast cells were biocompatible and exhibited lower levels of cytotoxicity when compared to the control, and, also, showed higher ALP activity. Waisi, et al. [180] showed that CNF composites with SiO_2_ particles have high surface area and flexibility. Waisi, Al-Jubouri and McCutcheon [180] did not evaluate the fibers’ biological characteristics, but Nekounam, et al. [181] produced CNF containing SiO_2_ nanoparticles by ES and observed that the incorporation of SiO_2_ nanoparticles increases hydrophilicity and improves cell attachment and viability. The carbon/silica nanofibers showed an increase in the proliferation rate of MG-63, indicating the strong osteoactive behavior of this compound.

In previous studies, Nekounam, et al. [182] also studied the influence of the incorporation of gold nanoparticles into CNF produced by two distinct methods: mixed electrospinning and simultaneously spun electrospinning/electrospraying. Indirect toxicity assays of MTT and *lactate dehydrogenase* (LDH) showed no significant toxicity that did not adversely affect cell proliferation. Samadian, et al. [117], in turn, deposited biomimetic HA crystals on electrospun CNF and studied the influence of the mineralization process. An increase in mechanical strength (*p* < 0.1) was reported, and the material transformed into one that was superhydrophilic and biocompatible. The produced compound induced higher new bone formation (61.3 ± 4.2%), when compared to the negative control group (*p* < 0.005).

In parallel with the development of ceramics for use in scaffolds aimed at bone regeneration, it has been observed, during the last two decades, the use of TII, such as Cu, Sr, Zn, Co, Si, and Bo, has the potential to increase bone formation and stimulate osteogenesis and angiogenesis [118,183]. Researchers have, also, observed [184,185] that some of these ions, such as Cu, Zn, and Ag, have anti-inflammatory and/or antibiotic action, which is very interesting with regard to bone grafts, in order to prevent inflammation and infections. In this context, studies [186] highlight that the use of these TII has advantages over the use of growth factors (organic molecules), such as the absence of decomposition risk, possibility of synergistic interaction between ions, and ability to be processed during the scaffold manufacture. Thus, doping/loading scaffolds with therapeutic ions has great potential for bone regeneration applications.

**Figure 11:**
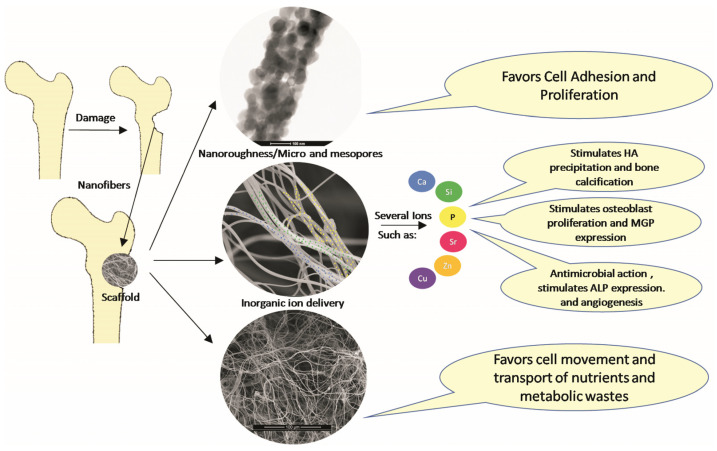
Schematic representation of the influence of ceramic nanofibers on the wound-healing process.

As highlighted before, the use of TII has been taking place since the beginning of the century, and has intensified in the last decade [116]. Examples of this include the doping of HA with Si [53,187], BG with Sr [188], and phosphate glasses with Mg [189]. In the last decade, the development of new bioactive glass compositions, containing ions such as Sr and Bo, has been observed [100], with it, also, being possible to highlight the addition of ions with bactericidal action, such as Zn and Sr, in a more comprehensive way in various bioceramics [76,190]. Figure 11 briefly demonstrates the important role of ceramic nanofibers and their various aspects, as well as the influence of TII in the bone-formation process.

In this sense, studies have focused on the production of ceramic nanofibers doped with therapeutic ions. Deliormanlı [191] prepared BG fibers (53% SiO_2_, 20% CaO, 6% Na_2_O, 12% K_2_O, 5% MgO and 4% P_2_O_5_) doped with cerium (Ce) and gallium (Ga) (1 to 5% by weight) through ES. Nanofibers exhibited good biocompatibility and the addition of Ce or Ga had no negative effect on the bioactivity in SBF. Moreover, tests on MC3T3-E1 pre-osteoblast cells using the MTT assay did not reveal nanofiber cytotoxicity in all the concentrations of the dopant element.

Weng, et al. [119] produced BG nanofibers (23.45% Si, 68.95% O, 2.28% P, 5.31% Ca; atom%) doped with Sr^2+^ and Cu^2+^ and observed that, when immersed in SBF, the presence of Sr promoted a fast formation of apatite crystals on the nanofibers’ surface, when compared to Cu-doped nanofibers. In in vitro cell culture, Sr significantly increased osteogenesis and suppressed osteoclastogenesis, while Cu promoted angiogenesis. These results corroborate with research that shows that Sr can help in bone homeostasis by stimulating osteoblasts, bone formation, and differentiation, as well as inhibiting osteoclastogenesis and bone resorption, in addition to exhibiting antimicrobial action [75,120,192]. Cu, in turn, may have an antibacterial effect and stimulate the proliferation of endothelial cells [193,194].

Tsai et al. [89] fabricated porous HA-CaO composite nanofibers loaded with Tetracycline (TC), presenting an average diameter of 461 ± 186 nm, which exhibited good drug-loading efficiency with the ability to delay the burst release of TC and maintain antibacterial activity, inhibiting bacterial growth for a seven-day period. Moreover, an outstanding drug-loading efficiency, a delay in the burst release of TC, and a maintenance in the antibacterial activity against Gram-positive bacteria *Staphylococcus aureus* and Gram-negative bacteria *Pseudomonas aeruginosa* were, also, observed in another work by Tsai, et al. [195], with Sr-substituted HA– CaO-CaCO_3_ nanofibers for over three weeks.

**Figure 12:**
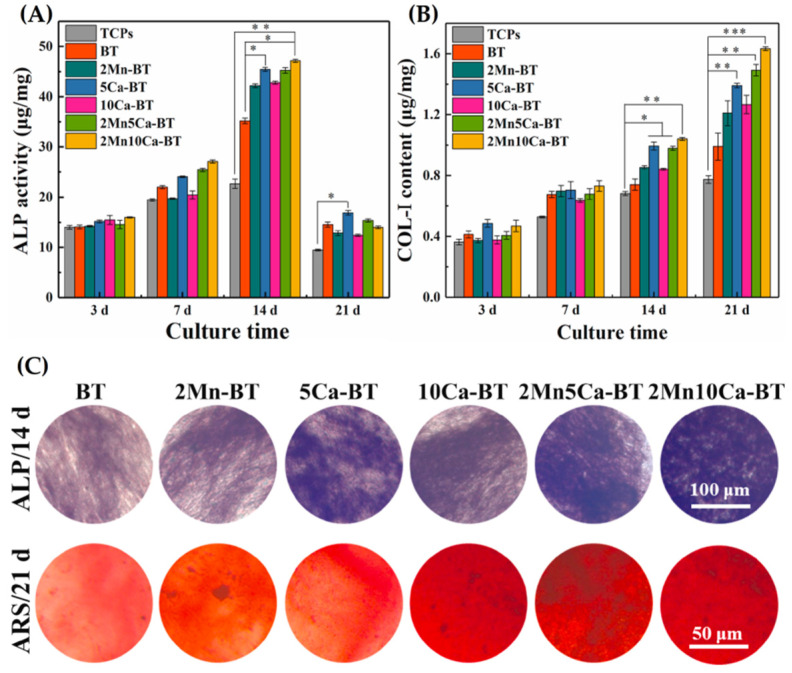
Evaluation of osteogenic differentiation of BMSCs cultured on doped and non-ion doped nanofibers compositions by an analysis of osteogenesis-related markers including: (**A**) quantitative analysis on ALP activity; (**B**) quantitative analysis on COL-I synthesis; (**C**) ALP staining and Alizarin red staining for calcium modules. * *p* < 0.05, significant; ** *p* < 0.01 and *** *p* < 0.001, highly significant (*n* = 4). Reprinted from [197], copyright (2021), with permission from Ceramics International.

Zheng et al. (2021) developed nanofibrous scaffolds made of BaTiO_3_ doped with Ca^2+^ and Mn^4+^ by ES. After calcination at 1000 °C, the nanofibers exhibited a d_33_ (piezoelectric coefficient) close to that of native bone. This study also indicated that doping with Ca can accelerate the degradation rate of BaTiO_3_, while doping with Mn can reduce this degradation rate. From Figure 12, it can be seen through quantitative analysis (ALP, COL-I) and smeared color depth (ALP, calcium modulus), that, when compared to TCPs, marker expressions were higher for cells grown in doped nanofibers. In addition, all ion-doped BaTiO_3_ nanofibers exhibited a greater ability to accelerate cell differentiation. In addition, BaTiO_3_ nanofibers co-doped with Mn^4+^ (2 mol%) and Ca^2+^ (10 mol%) did not exhibit any cytotoxicity and achieved the greatest ability to increase osteogenic differentiation of BMSCs, corroborating with studies [121,196] that showed that Mn and Ca doping do not promote toxicity, once both elements are inherent to human bone, in addition to being effective in promoting osteogenesis.

Considering what has been exposed about ceramic nanofibers for bone regeneration applications, Table 2 shows important information about the main groups of ceramic nanofibers used for bone regeneration and their most relevant biological properties, mentioning some relevant studies addressing this subject over the last decade.

## 4. Future Perspectives

Different types of biomaterials made of ceramic nanofibers have been studied for wound healing and bone tissue regeneration, with numerous advances in these materials’ development. Despite that, there are still limited data available in the literature addressing ceramic nanofiber application in regenerative medicine. This fact is related to the difficulty of the usual techniques for the production of nanofibers, notably the production volume, in addition to the fact that many of the ceramic fibers produced by these techniques have high fragility, which makes it impossible to produce materials with the necessary strength for handling and application such as a scaffold or a membrane.

Despite being brittle, scaffolds made from fibrous bioceramic materials are an excellent alternative for the application in biomedical tissue engineering; when compared to polymeric scaffolds, they have the ability to form an interface with living tissue through physical and chemical interactions, exhibiting good bioactivity. A majority of the studies involving the production of nanofibrious ceramic scaffolds observed an association between the inherent characteristics of the nanometric character of the microstructure (fiber topography, nanofiber arrangement, size, pore distribution, etc.) and the chemical–biological properties of the materials, often with a synergism between them, which usually implies outstanding cell adhesion, proliferation, and differentiation in in vitro tests.

Among the widely used ceramic materials, the use of SiO_2_ nanofibers can be highlighted due to their outstanding mechanical strength, large specific surface area, and good biocompatibility. CaP nanofibers are also widely used in several biomedical applications, due to their biocompatibility, bioactivity properties, and good osteoconduction and osseointegration characteristics.

Recent studies have demonstrated that the incorporation of inorganic therapeutic agents in nanofibrous ceramic systems is one of the new horizons of the regenerative medicine to improve biocompatibility, biodegradability, antimicrobial activity, wound-healing capacity, and bone -regeneration action. 

## Figures and Tables

**Table 1:** Summary information about ceramic nanofibers for wound healing application.

Nanofiber	Method	Composition (mol)	Physical–Chemical Properties	Diameter (nm)	Biological Properties	Reference
Silica hybrids	Electrospinning	Sílica (SiO_2_) Sílica (SiO_2_)–Bioactive glass (58% SiO_2_, 38% CaO, 4% P_2_O_5_)	Withstand autoclave sterilization Porosity: 93.2%	107–500	Non-cytotoxic, biocompatible, it facilitates the homogeneous growth of floclayer-type carbonated hydroxyapatite within a short period of immersion. Rapid cell growth with specific functions of hepatocytes per volume of substrates. They promote an increase in the hydrophilicity of the material, improving cell adhesion.	[20,66]
Hydroxyapatite hybrids	Electrospinning	Hydroxyapatite (Ca_10_(PO_4_)_6_(OH)_2_)–Silica (SiO_2_)	Surface area: 6.57 m^2^/g Pore volume: 0.025 cm^3^/g	110	Non-cytotoxic, biocompatible, bioactive, they have a high percentage of viability in a fibroblast lineage, stimulate cell growth, serve as cell support and allow cells to anchor. They promote the reduction in incision inflammation in vivo test after six weeks of surgical intervention.	[74]
Silver-containing nanofiber	Electrospinning	Silica (SiO_2_)-Silver (0.05, 0.1 and 0.15 Ag) Silver- Bioactive glass (2% B_2_O_3_, 68–69% SiO_2_, ~1 × 10^−3^ Ag_2_O, 29–30% CaO)	Properties not informed	200–390	They inhibit the proliferation of Escherichia coli with a long-term antibacterial effect, providing antibacterial potential to the wound bed. Non-cytotoxic, promoting cell growth over a wide concentration range. They allow the loading of drugs such as Tetracycline (TC) and have the ability to delay the release of TC and maintain antibacterial activity, inhibiting bacterial growth for a period of seven days.	[67,72,88]
Calcium-containing nanofiber	Electrospinning	Silica (100-X% SiO_2_)–Calcium (X% CaO), with X: 0, 20, 30, 40	Surface area: 40–43.1 m^2^/g	210–340	Increases the production of human vascular endothelial growth factor (VEGF) in a human dermal fibroblast cell line (CD-18CO) and promotes improved wound healing when compared to control.	[68]
Boron-containing nanofiber	Electrospinning	Bioactive glass–Boron (2% B_2_O_3_, 68–69% SiO_2_, 29–30% CaO)	Properties not informed	200–900	Higher wound healing rates after 24 h of testing. The presence of boron promoted healing of 82% and increased cell proliferation.	[88]
Cobalt-containing nanofiber	Electrospinning	Bioactive glass–Cobalt (50% SiO_2_, 24% Na_2_O, 24% MgO, 2% CoO)	The ability to act as both a network modifier and a network former	1000	They provided more sustained ion release compared to bioactive glass particles alone. Exposure of fibroblasts to the conditioned medium of these composites did not have a deleterious effect on metabolic activity, but the cobalt-containing glasses stabilized HIF-1α and caused significantly increased expression of VEGF (not observed in controls without Co).	[69]
Copper-containing nanofiber	Electrospinning	Borate bioactive glass- copper (6% Na_2_O, 8% K_2_O, 8% MgO, 22% CaO, 54% B_2_O_3_, 2% P_2_O_5_, 3% CuO)	Thermal stability	0.4–1.2 μm	Promising ability to stimulate angiogenesis and heal full-thickness skin defects.	[96]

**Table 2:** Summary information about ceramic nanofibers for application in bone tissue regeneration.

Nanofiber	Method	Composition (mol)	Physicochemical Properties	Diameter (nm)	Biological Properties	Reference
Calcium Phosphate	Electrospinning, Solution Blow Spinning	Hydroxyapatite (Ca_10_(PO_4_)_6_(OH)_2_) β-Tricalcium phosphate (Ca₃(PO₄)₂) Hydroxyapatite (Ca_10_(PO_4_)_6_(OH)_2_)-Silica (SiO_2_) Hydroxyapatite (Ca_10_(PO_4_)_6_(OH)_2_)–CaO Hydroxyapatite-Calcium (66.3% Ca_10_(PO_4_)_6_(OH)_2_), 21.1% CaO, 12.6% CaCO_3_)	Low strength and fracture toughness Surface area: 6.57–8 m^2^/g Pore volume: 0.025 cm^3^/g Pore size: 15.75–25 nm	100–460	High bioactivity, non-cytotoxic, and good biocompatibility, in addition to having good drug control release properties.	[31,56,74,89,122,195]
Bioactive glass	Electrospinning, Solution Blow Spinning, Template-Assisted Sol–Gel	Binary glass (60% Si, 40% Ca)	Surface area: 144.60–579 m^2^/g Porosity: 63.8% Pore size: 3.5–45 nm Pore volume: 0.21 cm^3^ g^−1^	16–358	Excellent biocompatibility, high bioactivity in SBF, high ALP activity, good degradation rate, promotes cell adhesion, and accelerates osteoblast proliferation and differentiation.	[32,55,154,156]
Wollastonite	Electrospinning, Hydrothermal Synthesis	β-wollastonite (β-CaSiO_3_) Wollastonite (CaSiO_3_)–Silica (SiO_4_)–Zinc (10% Zn)	High bending strength of 145.70 ± 2.74 MPa Porosity: 9.5–22.8%	10–500	Excellent bioactivity, good osteogenic differentiation of mesenchymal stromal cells, ability to release bioactive, and slowly degradable ions in inducing bone regeneration.	[128,164,170]
Hybridized carbon	Electrospinning, electrospinning/electrospraying	Carbon-Bioactive glass (89.65% C, 7.61% O, 2.28% Si, 0.10% P, 0.35% Ca) Carbon-Silica (5–10% SiO_2_) Carbon–Gold (1–2.5–5% Au) Carbon-Hydroxyapatite (34% C, 23% O, 11% P 32% Ca)	Higher dissolution rate High surface area and flexibility Porosity: 76%	190–320	Rapid cell proliferation and differentiation (indicating a strong osteoactive behavior), high ALP expression, biocompatible, and low level of cytotoxicity.	[54,117,179,181,182]
Therapeutic ions-containing nanofiber (Ce, Ga, Sr, Cu, Ca and Mn)	Electrospinning	Hydroxyapatite–Calcium (96.1% Ca_10_(PO_4_)_6_(OH)_2_), 1.4% CaO, 2.5% CaCO_3_)–Strontium (30% Sr) Bioactive glass (53% SiO_2_, 6% Na_2_O, 12% K_2_O, 5% MgO, 20% CaO e 4% P_2_O_5_) -Cerium-gallium (1–5% Ce and Ga) Barium titanate (BaTiO_3_)–Calcium-Manganese (10% Ca, 2% Mn)	Piezoelectricity, ion release and degradation behaviors. Pore size: 20–25 nm	103–582	Good biocompatibility, showed no cytotoxicity, improving bioactivity by promoting the activity of osteoblastic and endothelial cells, and inhibiting the formation of osteoclasts or bone resorption cells.	[119,191,195,197]
